# Resveratrol abrogates the Temozolomide-induced G2 arrest leading to mitotic catastrophe and reinforces the Temozolomide-induced senescence in glioma cells

**DOI:** 10.1186/1471-2407-13-147

**Published:** 2013-03-22

**Authors:** Eduardo C Filippi-Chiela, Marcos Paulo Thomé, Mardja Manssur Bueno e Silva, Alessandra Luíza Pelegrini, Pitia Flores Ledur, Bernardo Garicochea, Lauren L Zamin, Guido Lenz

**Affiliations:** 1 Department of Biophysics, Universidade Federal do Rio Grande do Sul (UFRGS), Rua Bento Gonçalves, 9500, Prédio 43431 – Lab. 107, Porto Alegre, RS CEP 91501-970, Brazil; 2 Center of Biotechnology, Universidade Federal do Rio Grande do Sul (UFRGS), Porto Alegre, RS, Brazil; 3 Universidade Federal da Fronteira Sul (UFFS), Cerro Largo, RS, Brazil; 4 Hospital São Lucas, Pontifícia Universidade Católica do Rio Grande do Sul (PUCRS), Porto Alegre, RS, Brazil; 5 Oncology Center - Hospital Sirio Libanes, Sao Paulo, SP, Brazil

**Keywords:** Glioblastoma, Resveratrol, Temozolomide, Autophagy, Mitotic Catastrophe, Senescence

## Abstract

**Background:**

Temozolomide (TMZ) is the most widely used drug to treat glioblastoma (GBM), which is the most common and aggressive primary tumor of the Central Nervous System and one of the hardest challenges in oncotherapy. TMZ is an alkylating agent that induces autophagy, apoptosis and senescence in GBM cells. However, therapy with TMZ increases survival after diagnosis only from 12 to 14.4 months, making the development of combined therapies to treat GBM fundamental. One candidate for GBM therapy is Resveratrol (Rsv), which has additive toxicity with TMZ in several glioma cells *in vitro* and *in vivo.* However, the mechanism of Rsv and TMZ additive toxicity, which is the aim of the present work, is not clear, especially concerning cell cycle dynamics and long term effects.

**Methods:**

Glioma cell lines were treated with Rsv and TMZ, alone or in combinations, and the induction and the role of autophagy, apoptosis, cell cycle dynamics, protein expression and phosphorylation status were measured. We further evaluated the long term senescence induction and clonogenic capacity.

**Results:**

As expected, temozolomide caused a G2 cell cycle arrest and extensive DNA damage response. Rsv did not reduced this response, even increasing pATM, pChk2 and gammaH2Ax levels, but abrogated the temozolomide-induced G2 arrest, increasing levels of cyclin B and pRb(S807/811) and reducing levels of pWee1(S642) and pCdk1(Y15). This suggests a cellular state of forced passage through G2 checkpoint despite large DNA damage, a scenario that may produce mitotic catastrophe. Indeed, the proportion of cells with high nuclear irregularity increased from 6 to 26% in 48 h after cotreatment. At a long term, a reduction in clonogenic capacity was observed, accompanied by a large induction of senescence.

**Conclusion:**

The presence of Rsv forces cells treated with TMZ through mitosis leading to mitotic catastrophe and senescence, reducing the clonogenic capacity of glioma cells and increasing the chronic effects of temozolomide.

## Background

More than 20,000 cases of malignant tumors of the Central Nervous System are diagnosed every year in the United States [http://www.cbtrus.org] [1]. Of these, more than half are Glioblastomas (GBM), which is the most malignant subtype and are normally treated with surgery, followed by radiotherapy and chemotherapy with Temozolomide (TMZ) [2,3]. However, TMZ therapy produces only modest increase in survival, maintaining GBM as a cancer with a median survival of about one year after diagnosis and causing over 10,000 deaths per year only in the USA. Therefore, treatment of GBM remains one of the hardest challenges to be tackled by oncotherapy [4].

TMZ is a cytotoxic imidazotetrazine that leads to the formation of O^6^-methylguanine, which mismatches with thymine in subsequent DNA replication cycles. This was described as leading to several cellular outcomes, such as apoptosis [5,6], autophagy [7]**,** mitotic catastrophe and senescence-like events [5] in GBMs. In most cells, TMZ produces cell cycle arrest at G2/M through activation of ATM/ATR-Chk1/2 [8]. Chk1/2 can activate Wee1, the kinase that phosphorylates Cdk1 at the inhibitory tyrosine 15 site, whereas it can inhibit CDC25A, the phosphatase responsible for dephosphorylating this site [9], thus leading to an arrest before mitosis. Activation of G2 checkpoint acts primarily as a prosurvival mechanism that gives time to the cells to repair their DNA. Impeding the cell cycle arrest in DNA-damaged cells normally leads to cell death by mitotic catastrophe (MC) [5], a failure caused by mitosis entry even in the presence of DNA damage or checkpoint activation [10,11]. Some cancer types, such as GBM, are intrinsically resistant to apoptosis and may be more sensitive to other mechanisms of cell death, such as autophagy, senescence and MC [12,13].

Resveratrol (Rsv) has several beneficial properties in age-associated chronic diseases, diabetes and cardiovascular diseases [14] and is neuroprotective in neurological conditions such as ischemia and hypoxia [15]. On the other hand, Rsv is cytotoxic to several types of malignant cells, such as colon cancer [16], breast cancer [17], melanomas [18], leukemia [19] and prostate cancer [20]. In GBM, it inhibits cell growth and causes cell death through mechanisms that include autophagy [21,22], apoptosis [23] and senescence [24]. Rsv exerts its cytotoxic and cytostatic effects through specific cell cycle modulation in several cancer types, including S-phase arrest in ovarian cancer [25] and medulloblastoma cells [26], G1 arrest in prostate cancer [27] and melanoma [28] and S/G2 arrest in leukemia cells [29].

Rsv was described to act synergistically with TMZ on apoptosis, accompanied by a decrease of TMZ-induced cytoprotective autophagy and a decrease of reactive oxygen species (ROS). This effect was mimicked by the antioxidants vitamin C and tiron, suggesting an effect mediated primarily by ROS [30]. *Yuan et al.* showed, in turn, that Rsv increased the TMZ-induced G2 cell cycle arrest in SHG44 glioma cells, accompanied by an increase in ROS production, leading to AMPK activation and mTOR inhibition, triggering apoptosis through the reduction of the antiapoptotic protein Bcl-2 [31]. However, the mechanism of action of the cotreatment is far from clear, and important mechanisms, such as cell cycle dynamics and long term effects of cotreatment *in vitro* were not evaluated, which may be fundamental to plan *in vivo* strategies.

Here we show that Rsv potentiates the cytotoxic effect of TMZ in human GBM cells by increasing DNA damage response (DDR) while blocking the TMZ-induced cell cycle arrest leading to MC and, in the long term, to senescence and reduction in clonogenic survival.

## Methods

### Reagents

TMZ (3,4-dihydro-3-methyl-4-oxoimidazo [5,1-d]-as-tetrazine- 8-carboxamide), Rsv, 3-methyladenine (3MA) and the fluorescent dye acridine orange (AO) were purchased from Sigma-Aldrich Chemical Co. (St. Louis, MO, USA). TMZ and Rsv were dissolved in dimethyl sulfoxide (DMSO) (Acros Organics, NJ, USA). 3MA and AO were dissolved in water. All culture materials were obtained from Gibco Laboratories (Grand Island, NY, USA).

### Cell culture and treatments

Human GBM cell lines U87-MG (p53^wt^, PTEN^mut^, p14ARF/p16^del^, low MGMT levels), U-138 MG (p53^mut^, PTEN^mut^, p14ARF/p16^del^, high MGMT levels) and U251 (p53^wt^, PTEN^null^, p14ARF/p16^del^, low MGMT levels), described hereafter only as U87, U138 and U251, were obtained from American Tissue Culture Collection (ATCC, Rockville, MD). Cell lines were cultured in DMEM low glucose, while primary cultures were maintained in DMEM high glucose, both supplemented with 10% fetal bovine serum (FBS), 1% penicillin/streptomycin and 0.1% amphotericin B at 37°C and 5% CO_2_ in a humidified incubator. The inhibitor 3-MA was used at he concentration of 2 mM, in a pre-incubation of 1 h before the treatments with Rsv and TMZ. The concentration of the vehicle DMSO did not exceed 0.5% (v/v). Cells were counted in a hemocytometer and viability was accessed by measuring PI incorporation as described [32]. Primary GBM culture was established from a biopsy of a GBM tumor following the ethical procedures approved by the Ethical Committee of PUC-RS number 07/03562.

### Detection and quantification of autophagy

*Autophagosome formation*: cells were transfected with the expression vector pEGFP-LC3 (Microtubule-associated protein 1 light chain 3 (MAP1-LC3), which localizes at the autophagosome membranes after processing [33]). Cells were imaged with a Zeiss Axiovert 200, using the 40x objective and at least 100 green cells per treatment were counted and the percentage of cells with at least 5 clear green dots in the cytosol was determined [34].

*Acidic vacuolar organelles (AVOs) quantification*: acridine orange (AO) is a marker of AVOs that fluoresces green in the whole cell except in acidic compartments (mainly late autophagosomes), where it fluoresces red. Cells were plated at 2 × 10^4^ cells per well in a 24-wells plate, followed by treatments as indicated. After this, cells were incubated with 2.7 μM of AO for 15 min at room temperature, followed by visualization in a fluorescence microscope. Images were analyzed using Image J software. To quantify the percentage of cells with AVOs (i.e. red marked cells) and the intensity of red fluorescence (i.e. the intensity of AVOs formation), treated cells were detached from the plate, marked with AO as cited above and analyzed by flow cytometry, using a flow cytometer GUAVA EasyCyte and GUAVA software ExpressPlus (Guava Technologies, Hayward, CA).

### Annexin-V staining

Apoptosis induction was quantified by Annexin V-FLUOS Apoptosis Kit (Roche, Germany) according to manufacturer’s instructions with minor modifications, as described [24].

### Cell cycle

For cell cycle analysis, cells were plated at 2 × 10^4^ cells per well in a 24-wells plate, followed by treatments as indicated. After treatments, cells were harvested and fixed in ice-cold ethanol 70% (v/v in PBS) for at least 2 h. Fixed cells were washed with PBS and marked with a solution containing 50 μg/ml PI, 0.1% Triton X-100 and 50 μg/mL RNAse for 30 min, in the dark, at room temperature. Marked cells were analyzed using the flow cytometer GUAVA EasyCyte software to evaluate DNA content of cells and, thus, cell cycle distribution of samples.

### Comet assay

TMZ, Rsv and cotreatment-induced DNA damage was quantified using the alkaline comet assay, as described by *Singh et al.*, with minor modifications [35-37]. Cells were plated at 5 × 10^4^ cells per well in a 24-wells plate, followed by treatments for 20 and 48 h, as indicated. Cells were embedded in 0.75% low-melting agarose and placed onto a glass microscope slide pre-coated with a thin layer of 1% normal melting point agarose. Slides were then incubated in ice-cold lysis solution [2.5 M NaCl, 10 mM Tris, 100 mM EDTA, 1% Triton X-100 and 10% DMSO, pH 10.0] at 4°C for at least 1 h. After, slides were incubate with fresh alkaline buffer (300 mM NaOH, 1 mM EDTA, pH. 13.0) and followed by electrophoresis. Slides were then neutralized (0.4 M Tris, pH 7.5), washed with water, and stained using a silver staining protocol as described by *Nadin et al.*[38]. One hundred nuclei were scored blindly according to the amount of DNA present in the tail and the tail length. Each nuclei received an arbitrary value range from 0–4 (0, undamaged; 4, maximally damaged) [39], and 100 nuclei per slide were evaluated.

### Western blot

Analysis of protein expression and phosphorylation was performed as described previously with minor modifications [15,24]. Primary antibodies used were: cyclin D1 (1:1000), phospho-Rb (S807/811)(1:1000), phospho-Cdk1 (Tyr15)(1:1000), cyclin B (1:250), phospho-ATM (Ser1981)(1:1000), phopho-Chk2 (T68)(1:1000), gammaH2AX (1:1000), phospho-Wee-1 (S642) (1:500) and phospho-H3 (Ser10) (1:1000) (Cell Signal ling, Beverly, MA). Optical density of the bands was obtained with Bio-Rad software (Quantity One; Hercules, CA).

### Clonogenic assay

For clonogenic assay, cells were treated with Rsv, TMZ or cotreatment for 48 h, followed by medium removal. Cells were washed twice with PBS, harvested and plated at a density of 10^2^ cells/well in a 6-wells plate. After 14 days, colonies were fixed with methanol, followed by staining with 0.1% crystal violet. The number of colonies was counted and single colonies were photographed for analysis.

### SA-beta-gal assay

For senescence measurement, cells were treated with Rsv, TMZ or cotreatment for 48 h, followed by medium removal. Cells were washed twice with PBS and replated at a density of 20 × 10^3^ cells/well, in a 12-wells plate. After seven days, cells were tested for senescence as described [40], with minor modifications. Briefly, cells were washed with PBS, fixed with 2% paraformaldehyde for 30 min at room temperature and incubated with fresh SA-beta-gal staining solution (1 mg/mL X-gal (Sigma), 40 mM citric acid/sodium phosphate (pH 6.0), 5 mM potassium ferrocyanide, 5 mM potassium ferricyanide, 150 mM NaCl, and 2 mM MgCl) for 8–12 h at 37°C. Then, cells were marked with a solution containing 300 nM DAPI and 0.1% triton X-100 (v/v in PBS) for 30 min at room temperature. Results are presented as ratio of SA-beta-gal-positive cells to total cells.

### Nuclear Morphometric Analysis (NMA)

The analysis of nuclear morphometry was performed using a tool recently developed by our group [41]. Briefly, cells were treated as described in SA-beta-gal assay and, at day 7, cells were fixed with 2% paraformaldehyde (v/v in PBS) for 30 min at room temperature, and kept in PBS. Next, fixed cells were marked with a solution containing 300 nM DAPI and 0.1% triton X-100 (v/v in PBS) for 30 min at room temperature, followed by quantification of the images obtained with DAPI staining using the Software Image Pro Plus 6.0 (IPP6 - Media Cybernetics, Silver Spring, MD) or Image J plugin available at http://www.ufrgs.br/labsinal/nma. Data is presented as a plot of Area *versus* Nuclear Irregularity Index (NII), which separates nuclei considering its morphometric phenotype. The percentage of normal, irregular, large and regular, large and irregular, small, small and regular and small and irregular nuclei were determined as described [41].

### DCF (dichlorofluorescein) assay

To measure the levels of reactive species, we performed the DCF assay. The fluorescein derivative DCF (Sigma-Aldrich) is a non-fluorescent compound which is converted to a highly fluorescent DCF upon oxidation by oxygen or nitrogen reactive species. To this, 5 × 10^4^ cells were plated in 24-well plates, followed by treatments as indicated. Cells were harvested, washed once with PBS 1× and incubated with 10 μM (in PBS 1×) for 30 min at 37°C prior to analysis by flow cytometry.

### Statistical analysis

Statistical analysis was conducted by ANOVA followed by SNK post-hoc test to multiple comparisons of at least three independent experiments for all experiments, except when indicated. ‘*p*’ value under 0.05 was considered significant. Analyses were performed using the GraphPadInstat software (GraphPad Software, San Diego, CA, USA).

## Results

### Rsv potentiates the cytotoxic effects of TMZ

**Figure 1:**
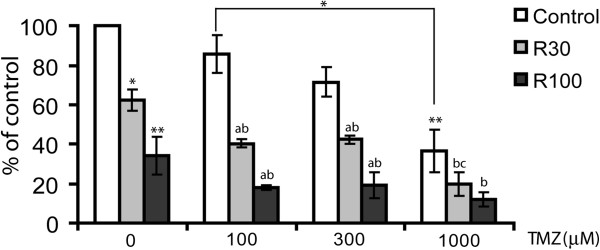
**Rsv potentiates the cytotoxic effects of TMZ in human glioma cells.** U87 glioma cells were treated with the indicated doses of Rsv (30 or 100 μM), TMZ or combinations for 48 h, followed by cell counting; control cells were considered 100%; *p<0.05 and **p<0.01, in relation to control; ^*a*^ p<0.05 and ^*b*^ p<0.01 in relation to TMZ and Rsv alone, respectively.

TMZ and Rsv individually reduced the number of glioma cells (Figure 1), as shown by others [6,7,24]. When given together, Rsv potentiated the cytotoxic effect of TMZ on human glioma cell lines U87, U138 and U251 (Figure 1 and Additional file 1: Figure S1a). A primary GBM culture which was resistant to TMZ was sensitive to Rsv and to RT (Additional file 1: Figure S1b). Rsv 30 μM plus TMZ 100 μM (hereafter referred to as RT) did not induce loss of membrane integrity, large morphological alterations, increase in annexinV/PI-staining or ROS when cells were treated for 48 h (Additional file 2: Figure S2), indicating that apoptosis, necrosis or a major imbalance in ROS levels are not involved in the reduction in cell number induced by RT.

### Rsv increases autophagy induced by TMZ, but autophagy does not affect acute cell death

**Figure 2:**
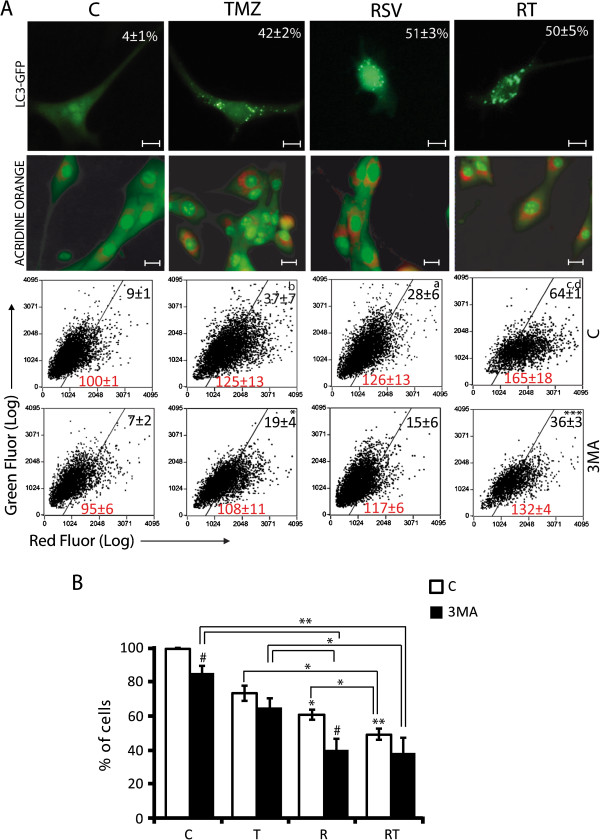
**Autophagy is not involved in the toxicity of RT cotreatment. (A)** U87 cells were pre-incubated with 3MA (2 mM) or vehicle (PBS) for 1 h before treatment with Rsv 30 μM, TMZ 100 μM or RT for 48 h. *Upper line* – LC3-GFP transfected cells; number = percentage of LC3-GFP-positive cells; scale bar: 10 μm; *second line* - representative images of AO-marked cells; scale bar: 20 μm; *third and fourth lines* – flow cytometry of AO-marked cells; numbers in the quadrants refer to average number of events (black) or mean intensity of red fluorescence (Red) ± SEM; ^*a*^ p<0.05, ^b^ p<0.01 and ^*c*^ p<0.001 in relation to control; ^*d*^ p<0.001 in relation to TMZ alone; * p<0.05 and *** p<0.001 in relation to treatments with the vehicle; **(B)** number of cells after pre-treatment with 3MA followed by treatment as in (a); control was considered 100%; * p>0.05; ** p>0.01, and # p>0.05 in relation to the same treatment without 3MA.

TMZ, Rsv or RT induced autophagy (Figure 2A, Additional file 1: Figure S1B and Additional file 3: Figure S3) both in primary GBM cells and cell lines, which can be partially inhibited with 3MA, an inhibitor of PI3K class III, a protein whose activity is necessary for the early formation of autophagosomes (Figure 2A). RT induced higher levels of autophagy in an additive manner with respect to Rsv and TMZ alone (Figure 2A, Additional file 1: Figure S1 and Additional file 3: Figure S3), however, an efficient inhibition of autophagy with 3MA did not reduce toxicity of treatments, even increasing the toxicity of Rsv, suggesting a small protective role of autophagy (Figure 2B).

### Rsv abrogates TMZ-induced cell cycle arrest

**Figure 3:**
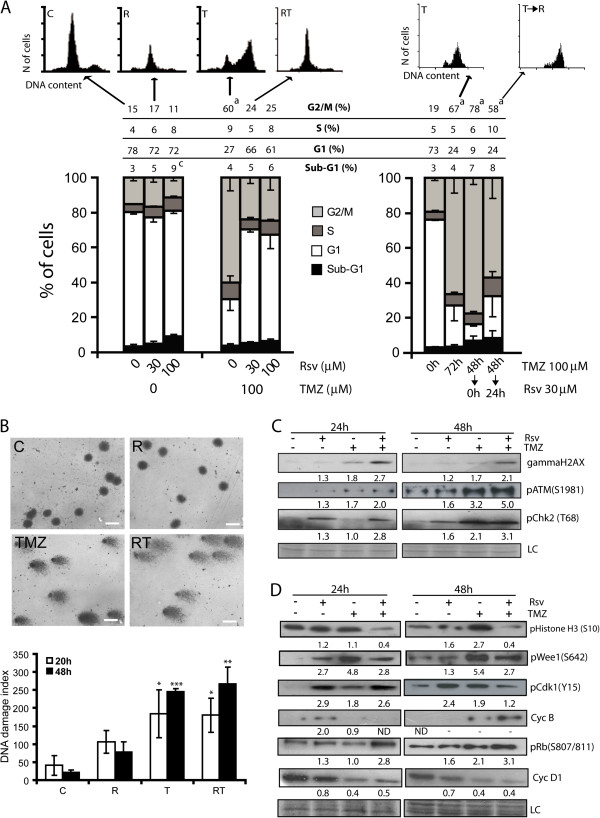
**Rsv abrogates TMZ-induced G2 cell cycle arrest accompanied by decrease of Cdk1(Y15). (A)** Cell cycle distribution of U87 cells treated with Rsv 30 μM, TMZ 100 μM or cotreatment T100+R30 for 48 h (left) TMZ for 72 h, or TMZ 48 h followed by 24 h in complete drug-free medium, or 24 h of Rsv 30 μM (right). Top: representative histograms of flow cytometry; numbers between the lines indicates the percentage of cells in each phase of cell cycle, as indicated; ^*a*^ p<0.001 and ^*c*^ p<0.05 in relation to control without drug; **(B)** DNA damage index measured by the comet assay in cells treated as in (a) for 20 and 48 h. **top panel –** representative images of nuclei after comet assay; scale bar: 10 μm; **bottom** – quantification of DNA damage index; **(C** and **D)** U87 cells were treated with Rsv 30 μM, TMZ 100 μM or cotreatment T100+R30 for 24 or 48 h, followed by western blotting using the indicated antibodies. Numbers indicate the band intensity in relation to control. LC – loading control (stained membrane); ND – not detected; * p<0.05, ** p<0.01 and *** p<0.001 in relation to control of the same time.

As expected, TMZ induced a robust G2/M cell cycle arrest in U87 cells, while Rsv did not alter cell cycle distribution after 48 h of treatment. Interestingly, the presence of Rsv completely abrogated TMZ-induced cell cycle arrest (Figure 3A - mid graph). The addition of Rsv right after a 48 h treatment with TMZ did not alter the arrest induced by TMZ, suggesting that Rsv inhibits rather than reverses the cell cycle arrest induced by TMZ (Figure 3A – right). In U251 cells, Rsv induced a significant S arrest and slightly reduced TMZ-induced G2/M arrest, although the effect is less clear due to the S phase-arrest induced by Rsv. In U138, Rsv induced a S-phase arrest, but no clear TMZ-induced arrest was observed, which may be due to the high expression of MGMT (methyl-guanine methyl transferase) in these cells [42] (Additional file 4: Figure S4).

Since Rsv modulates proteins involved on DNA repair and interacts directly with DNA *in vitro*[43], we asked whether Rsv was abrogating cell cycle arrest due to a block of TMZ-induced DNA damage or DDR signaling. Quantification of DNA damage by comet assay indicated that Rsv did not reduce the TMZ-induced DNA damage (Figure 3B) nor DDR signaling, as indicated by the phosphorylation levels of H2AX, ATM and Chk2 (Figure 3C). Actually, RT treatment induced higher levels of gammaH2AX, pATM and pChk2 when compared to single treatments.

DDR signals to mitosis by activating Wee1 and inhibiting CDC25A, which are, respectively, negative and positive regulators of Cdk1 (Cdc2) [9,44]. Wee1 phosphorylates whereas CDC25 dephosphorylates tyrosine 15 of Cdk1, and dephosphorylation of this site is required for the passage of G2 to M [45]. The increased levels of DDR signaling induced by RT (Figure 3C) does not translate into increased levels of pWee1, in which RT-treated cells have a lower level when compared to Rsv- or TMZ-treated cells, suggesting that Rsv acts as an inhibitor of the phosphorylation and activation of Wee1, leading to a reduced level of pCdk1 (Y15), (Figure 3D). Serine 10 of histone H3 is phosphorylated during mitosis and cells arrested at G2/M by TMZ showed an increase in the phosphorylation of this site whereas this was significantly reduced in RT treated cells. Surprisingly, levels of Cyclin B1, which has to be degraded for mitotic release, were found elevated in RT treated cells (Figure 3D). Noteworthy also is the observation that phospho(S807/811)-Rb (pRb), which is phosphorylated at the G1 to S transition [46] and dephosphorylated at the completion of M [47], is elevated with RT (Figure 3D). This reduction of pCdk1 (Y15) to mitotic permissive levels together with increased levels of Cyclin B1 leads to a molecular state which can lead to failed mitosis [48].

### RT induces mitotic catastrophe

**Figure 4:**
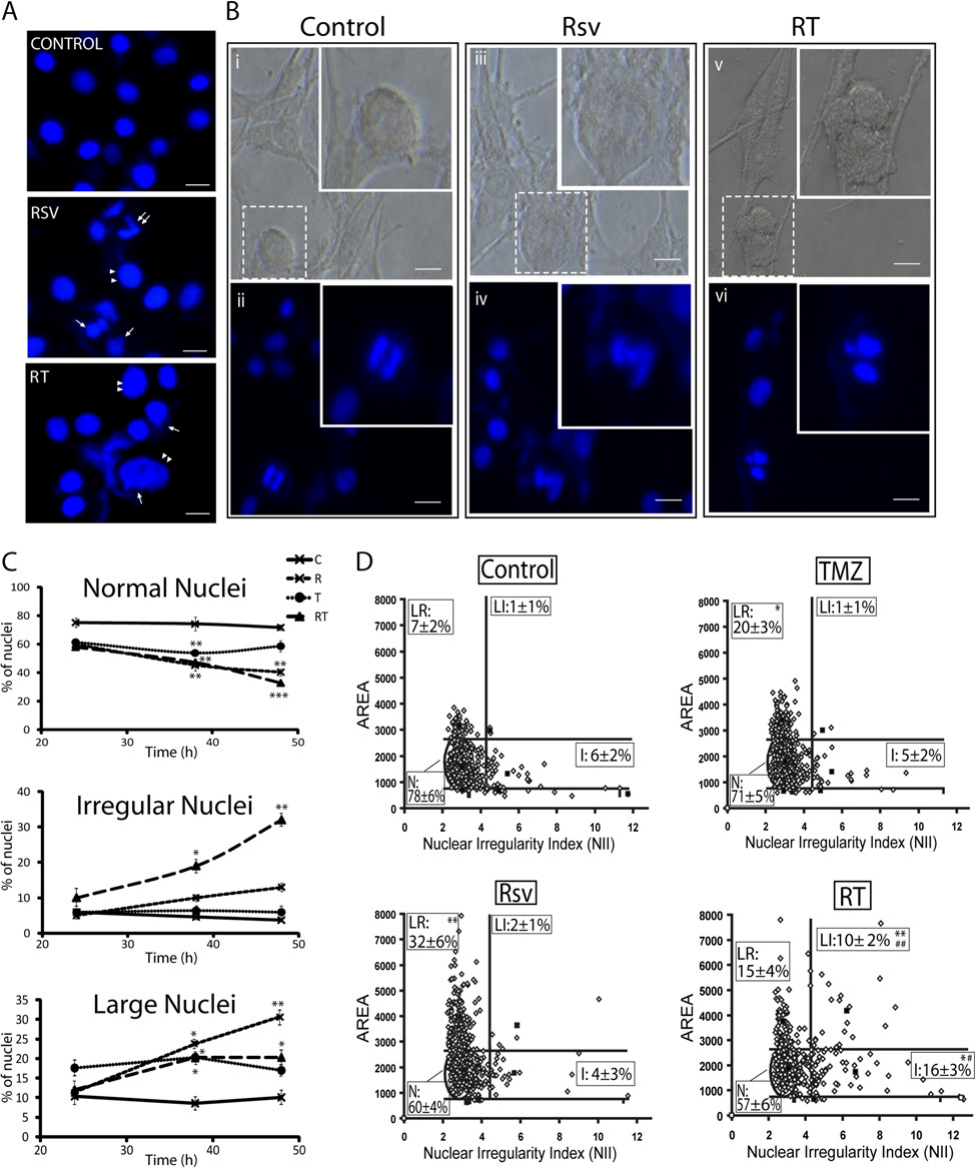
**Inhibition of TMZ-induced G2 arrest by Rsv leads to mitotic catastrophe.** U87 cells were treated with Rsv 30 μM, TMZ 100 μM or RT for 24, 38 and 48 h, followed by fixation and DAPI staining. **(A)** representative images of nuclei from cells treated with DMSO (control), Rsv for 38 h or RT for 48 h. Double arrows point to fragmented/irregular nuclei; single arrow points to micronuclei; double arrowheads point to enlarged nuclei; Scale bar: 6 μm; **(B)** representative visible and fluorescent images of nuclei from untreated cells showing a normal mitosis **(i and ii)**; an abnormal chromosome condensation and mitosis, with cellular enlargement, in a cell treated with RT for 48 h **(iii and iv)**; and a triple mitosis in a cell treated with Rsv for 48 h **(v and vi)** and. Scale bar: 10 μm; **(C)** direct counting of the percentage of nuclei presenting normal (top), irregular (mid) or large phenotype (bottom); *p<0.05, **p<0.01 and ***p<0.001 in relation to control; **(D)** DAPI-stained nuclei were analyzed for size and irregularity using the NMA tool, as described on material and methods, and the percentage of each nuclear type is shown. At least 200 nuclei in three independent experiments were analyzed in each data point; *p<0.05 and **p<0.01 in relation to control; ^#^p<0.05 and ^##^p<0.01 in relation to TMZ and Rsv alone.

Cells with DNA damage that bypass cell cycle checkpoints tend to undergo MC [10,49]. We observed that RT-treated cells presented alterations that suggest MC (Figure 4A and 4B), i.e. aberrant chromosome condensation and mitotic phenotype, micronuclei and nuclear fragmentation, and abnormal/triple mitosis.

We developed an objective morphometric analysis of nuclei size and irregularity [41], which showed that RT treatment increased the percentage of irregular nuclei, characteristic of MC, when compared to the individual treatments (Figure 4D), in agreement with direct counting of irregular nuclei (Figure 4C). It is also important to notice that the percentage of large nuclei, a feature of senescence induction, as previously described for another glioma cell line [24], was increased by TMZ, Rsv and RT treatments (Figure 4C and D).

### Senescence mediates the chronic effects of RT treatment

**Figure 5:**
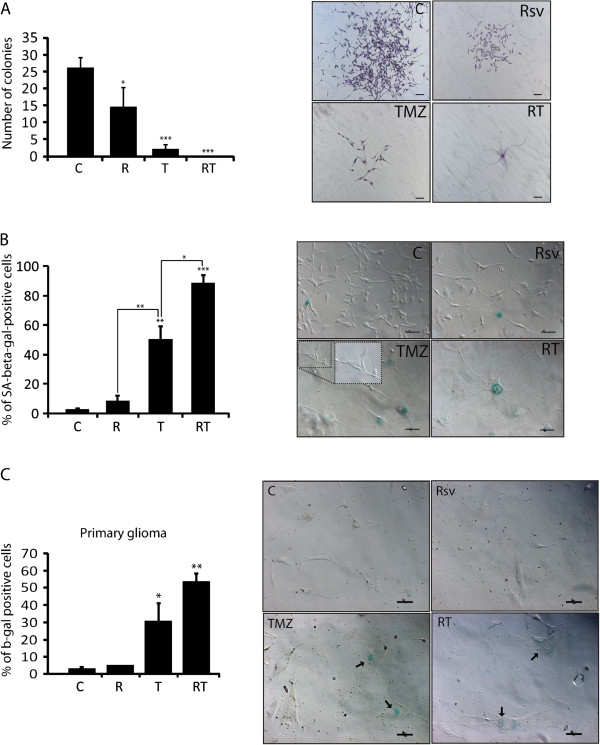
**Cotreatment of Rsv and TMZ reduces clonogenic growth and induces senescence in glioma cells.** U87 cells were treated with Rsv 30 μM, TMZ 100 μM or RT for 48 h, followed by two washes for drug removal. Then, **(A)** cells were plated for clonogenic assay; **left** – number of colonies after 14 days; **right** – representative images of colonies; scale bar: 100 μm; **(B)** cells were treated as above and plated for senescence measurement; **left** – percentage of SA-beta-gal-positive cells; **right** – representative images of treated cells; insert = SA-beta-gal-negative cells in TMZ-treated image. ** p<0.01 and *** p<0.001 in relation to control. **(C)** primary glioblastoma cells were treated as in above followed by growth for 7 days in DFM and analyzed by SA-beta-gal assay.

To test the long term effect of RT treatment, which more closely correlates with *in vivo* data [50], U87 cells were treated for 48 h followed by Drug-Free Medium (DFM). After fourteen days, Rsv and TMZ reduced the number of colonies formed in 40% and 90%, respectively, in relation to control (Figure 5A, left graph). Furthermore, colonies formed from Rsv or TMZ-treated cells were much smaller when compared to untreated colonies. In RT-treated cells, in turn, no colonies were observed, but only individual, senescent-like cells (Figure 5A, right images).

TMZ induced senescence in around 50% of cells after 7 days in DFM, with the remaining cells showing normal morphology (Figure 5B). These cells may be responsible for the colonies observed after TMZ treatment (Figure 5A). RT induced a much higher proportion of SA-beta-gal-positive cells than treatments alone in both U87 (Figure 5B) and in primary GBM cells (Figure 5C). SA-beta-gal-negative cells were not observed in RT treated cultures (Figure 5B, insert), suggesting that Rsv could reduce the appearance of TMZ resistance.

## Discussion

Despite the use of a multimodal therapy, the prognosis of GBM did not change in recent years [4]. Cytotoxicity of clinically achievable serum levels of TMZ, which are around 100 μM [51], was very small in the three glioma cell lines and, furthermore, the primary glioma tumor tested was resistant to TMZ, while Rsv was cytotoxic alone and potentiated the effect of TMZ in these cells. Therefore, drugs that enhance the effect of TMZ have been actively studied [52,53] and may represent an alternative strategy for combined chemotherapy.

The effect of Rsv in increasing TMZ-induced toxicity and autophagy occurred in all glioma cells tested, demonstrating that it does not involve the p53 pathway, since U251 and U138 cells are p53 mutant. The lack of correlation between autophagy and cytotoxicity in three GBM cell lines and the absence of reversion of the cytotoxicity by 3MA, despite a significant reduction in autophagy, suggest that this is not a central mechanism in the reduction in cell number. Indeed, autophagy induced by co-treatment seems to be protective rather than cytotoxic. In U138 cells, which have high levels of MGMT protein when compared to U251 and U87 cells, TMZ did not trigger G2-cell cycle arrest, while U87 and U251 cells arrested at G2 with TMZ, also suggesting a p53-independent arrest [5,54]. Furthermore, in agreement with our data in U87 cells, *Mhaidat et al.* showed that sensitivity of melanoma cells to TMZ was associated with MGMT status, G2 arrest and senescence entry, while no apoptosis was induced [55].

Rsv induced a transitory S-phase arrest [21] and a small increase in the phosphorylation of ATM, Chk2 and H2AX in U87 cells, while leading to a more prolonged S-phase arrest in U251 and U138 cells. Others also reported Rsv-induced S-phase arrest via DDR signaling [25]. Interestingly, Rsv induced an increase in nuclear size after 48 h of treatment in 32% of cells, an indication of senescence induction, but after 7 days in DFM no senescent cells remained, suggesting that the undamaged cells repopulated the well. On the other hand, damage induced by TMZ seems to be more long lasting, since 20% of large nuclei were observed after 48 h and 50% of cells were SA-β-gal positive after 7 days in DFM, suggesting that even in the absence of external TMZ, the damage induced in the 48 h of treatment was maintained. Important to notice that the combination of RT induced 25% of large and large irregular nuclei at 48 h and 90% of SA-β-gal positive after 7 days in DFM, suggesting that the presence of Rsv potentiates the long lasting damage that leads to senescence induced by TMZ.

Cell cycle arrest acts as a pro-survival mechanism, since it gives extra time for DNA repair [5]. This is supported by the effect of an inhibitor of Chk2, which enhances the toxicity of TMZ through induction of MC [56-59]. TMZ-induced G2/M cell cycle arrest was totally abrogated by Rsv in U87 cells, without altering DNA damage and ROS levels. However, RT treatment increased the levels of gammaH2AX, pATM and pChk2 in relation to the treatments alone. DNA damage links to the mitotic machinery through phosphorylation of CDC25 and Wee1 by Chks, inhibiting and activating these enzymes, respectively. This leads to phosphorylation of tyrosine 15 and consequent inhibition of Cdk1 [60]. Experiments in *Xenopus* indicate that xChk1 phosphorylates xWee1 on Ser549, which corresponds to Ser642 in hWee1 [60]. Phosphorylation of this site in hWee1 was shown to be important for 14-3-3 binding and activity, and Chk1 was among the kinases able to phosphorylate this site in HELA cells [61]. TMZ induced high levels of pWee1(S642), which were reduced by the cotreatment with Rsv, leading to a reduction in pCdk1 after 48 h of treatment, suggesting a permissive status for mitotic progression.

On the other hand, several molecular signs in RT treated cells suggest G2 cell cycle arrest or early mitosis: (1) high levels of pRb(S807/811), which is phosphorylated at the G1 to S transition [62] and dephosphorylated at the M to G1 transition [63]; (2) low levels of cyclin D1; (3) high levels of cyclin B1, which needs to be degraded for the completion of mitosis [48]; and (4) low levels of histone H3 S10 phosphorylation, which occurs in the early step of mitosis, being tightly correlated with chromosome condensation during mitosis [64]. This state of conflicted cell cycle signaling, mainly accumulation of cyclin B [65] and decrease of Cdk1(Y15) [66] was also observed in GBM cells treated with radiation or doxorubicin and fluorouracil [67,68] and was the underlying mechanism of MC induction. Indeed, we observed an increase of cells with MC features after RT treatment, which was not observed in the isolated treatments.

In summary, considering the effect of Rsv on TMZ-treated cells we noted that there was no effect on DNA damage. At the early steps of DDR signaling, γH2AX, pATM and pChk2, Rsv increased the levels induced by TMZ. On the other hand, effectors of DDR signaling that regulate the cell cycle were decreased by Rsv in TMZ treated cells, mainly pWee1, pCdk1 and Histone H3 phosphorylation, which are targets of Chk1/2. This indicates that Rsv somehow potentiates the early steps of DDR signaling while blocking the late stages, probably at the level of Chk1/2, which may be responsible for the lack of TMZ-induced arrest. Although the proportion of cells with nuclear irregularities was relatively low (26%) after 48 h, stretched out over the duration of a typical TMZ therapy, i.e. several weeks, this mechanism, together with senescence induction, may be responsible for the observed total reduction in clone formation after 14 days. Indeed, the multiple mechanism of action of TMZ plus Rsv cotreatment in glioma cells is therapeutically promising, since the induction of autophagy [7], senescence and mitotic catastrophe, rather than high level of apoptosis [69] seems to be important for the mechanism of action of TMZ on GBM cells.

In conclusion, Rsv increases the toxicity of TMZ in GBM cells through induction of multiple mechanisms, mainly inhibition of TMZ-induced G2/M arrest followed by induction of senescence and MC. The effects of the RT combination are more clearly observed with long term analysis, in which the combination totally abrogates clonogenic growth. Thus, development of combinations of drugs that induce multiple mechanisms of cell death and growth arrest deserves attention and has a high potential to be pre-clinically tested, due to the effectiveness to induce the disruption of tumor cells, thus, reducing the possibility of resistance and recurrence.

## Conclusions

In conclusion, our results showed that Rsv increases the toxicity of TMZ in GBM cells mainly through the inhibition of the G2/M arrest and induction of MC. Considering that almost 70% of GBM presented alterations on p53 pathway [70], it is clinically relevant that a combined therapy affects both populations, as was the case of the combination of Rsv and TMZ. In the same way, considering the primary therapeutic intervention to GBM, which includes chemo and radiotherapy, the observation that TMZ sensitizes GBM cells to radiation not through apoptosis, but through MC induction is also noteworthy [32]. Thus, development of combinations of drugs that induce MC, such as RT, deserves attention and should be tested clinically, due to the effectiveness of this mechanism to induce cell disruption.

## Abbreviations

GBM: Glioblastoma; TMZ: Temozolomide; Rsv: Resveratrol; MC: Mitotic catastrophe; RT: Resveratrol+temozolomide cotreatment; DFM: Drug-free medium; ROS: Reactive oxygen species; 3MA: 3-methyladenin; AO: Acridine orange.

## Competing interests

The authors declare that there are no conflicts of interest.

## Authors’ contribution

ECFC planed and performed the majority of the experiments and wrote the manuscript; MPT and MMBS participated in several of the experiments; ALP performed the experiments related to DNA damage and repair; PFL and BG performed the primary glioma cultures; LLZ contributed with the design of several experiments and GL supervised the study and wrote the manuscript. All authors read and approved the final manuscript.

## Pre-publication history

The pre-publication history for this paper can be accessed here:

http://www.biomedcentral.com/1471-2407/13/147/prepub

## Supplementary Material

Additional file 1: Figure S1 Autophagy and toxicity in other glioma cell. (a) U87, U138 or U251 cells were treated with Rsv 30 μM, TMZ 100 μM or RT for 48 h a, followed by cell number determination **(upper panel)** and marking with AO followed by flow cytometry to determine the percentage of AO-positive cells **(lower panel)**; **(b)** primary glioblastoma cells were treated as above and analyzed for cell number and AO staining after 48 h Numbers in the quadrants refer to average of events (black) or X-mean of AO red fluorescence intensity (red) ± SEM of three independent experiments; * p<0.05, ** p<0.01. Click here for file

Additional file 2: Figure S2 Apoptosis or ROS are not involved in RT toxicity. U87 cells were treated with Rsv 30 μM, TMZ 100 μM or RT for 48 h. After this, cells were **(a)** stained with 6 μM PI, to evaluate the membrane integrity and induction of necrosis - numbers indicate the percentage of positively marked cells (ratio of PI labeled cells/total cells) for at least 100 cells counted per treatment; scale bar: 100 μm; detail shows the morphology of treated cells. **(b)** Cells were treated as in (a) and marked with annexin V-FLUOS/PI and evaluated by flow cytometry. Numbers in quadrants represents the percentage of cells ± SEM of three independent experiments; **(c)** Cells were treated as in (a) and marked with DCFH to measure reactive oxidative species followed by flow cytometry. **Left** -% in relation to control of DCFH intensity; **right** – graphs of DCFH staining from Guava Software; n=2. Click here for file

Additional file 3: Figure S3 Autophagy is not directly involved on toxicity of the cotreatment of Rsv and TMZ. (a) Representative images of U87 human GBM cells transfected with the plasmid pRGFP-LC3 and treated with Rsv 30 μM, TMZ 100 μM or R30+T100 for 48 h; white arrows: cytosolic green dots representing LC3-GFP marked autophagosomes; scale bar: 10 μm; **(b)** percentage of cells that presented more than five well-defined cytosolic green dots, for each treatment; ^*a*^ p<0.01 and ^*b*^ p<0.001 in relation to control; **(c)** cells were treated as in (A), marked with AO and the percentage of cells positively marked to acridine orange (i.e. red marked cells) were evaluated by flow cytometry after 4, 24 and 48 h; ^*a*^ p<0.05 and ^*b*^ p<0.01 in relation to control. Click here for file

Additional file 4: Figure S4 Rsv abrogates TMZ-induced arrest in G2/Min U251 cells, while U138 is resistant to TMZ-induced arrest. Cell cycle distribution of U251 and U138 cells treated with Rsv 30 μM, TMZ 100 μM or RT, for 48 h. Representatives histograms are shown on the top of figure of flow cytometry; numbers between the lines indicates the percentage of cells in each phase of cell cycle, as indicated; cells were treated as cited above, followed by fixation as described on material and methods section, staining with 6 μM PI and flow cytometry to determination of DNA content; * p<0.05; ** p<0.01. Click here for file
