# Supplementary figures and images for: Resveratrol abrogates the Temozolomide-induced G2 arrest leading to mitotic catastrophe and reinforces the Temozolomide-induced senescence in glioma cells
Source: BMC Cancer. 2013 Mar 22;13:147. doi: 10.1186/1471-2407-13-147 (PMC3635906; doi:10.1186/1471-2407-13-147)

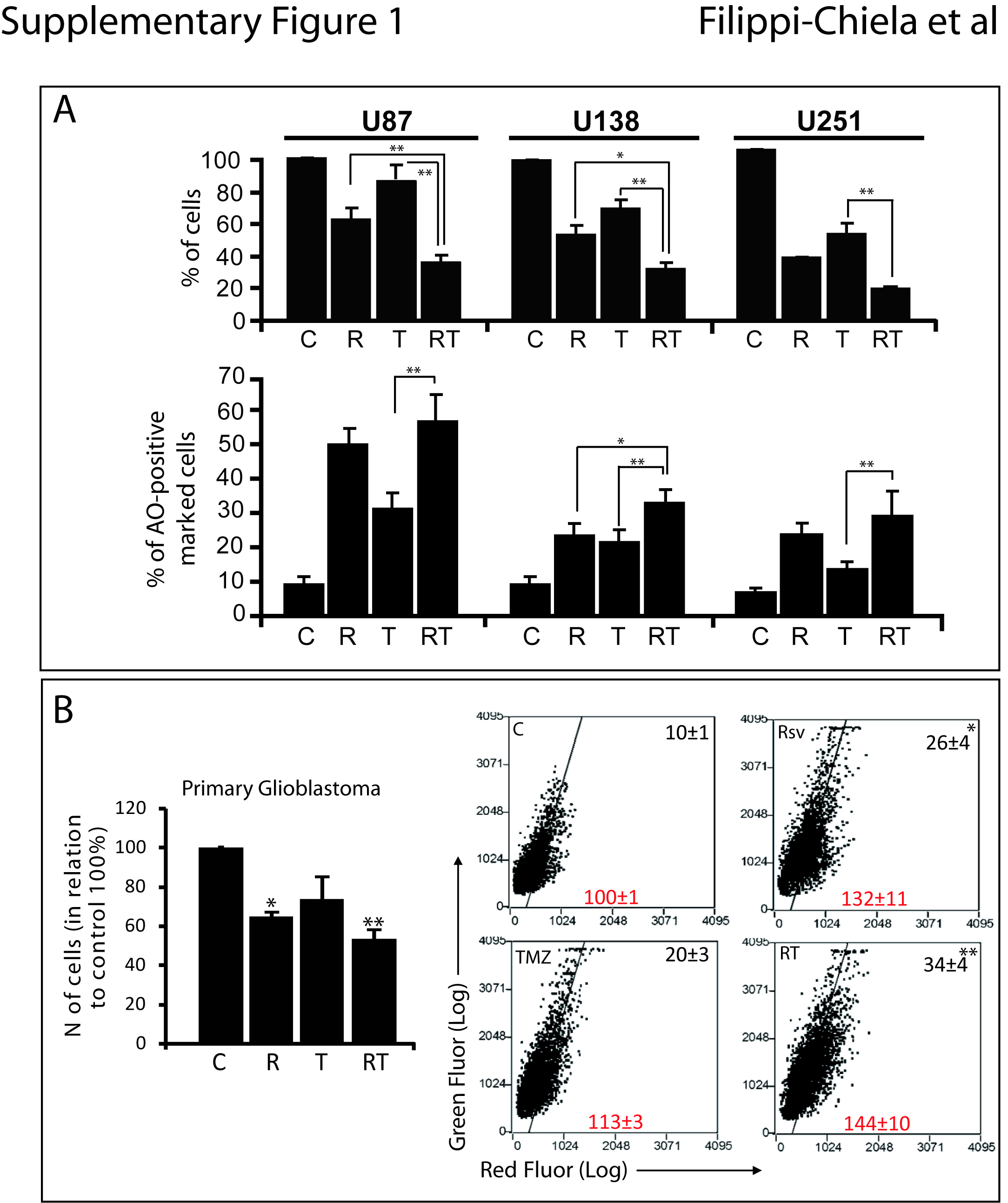

Supplement: Additional file 1: Figure S1 — Autophagy and toxicity in other glioma cell. (a) U87, U138 or U251 cells were treated with Rsv 30 μM, TMZ 100 μM or RT for 48 h a, followed by cell number determination (upper panel) and marking with AO followed by flow cytometry to determine the percentage of AO-positive cells (lower panel); (b) primary glioblastoma cells were treated as above and analyzed for cell number and AO staining after 48 h Numbers in the quadrants refer to average of events (black) or X-mean of AO red fluorescence intensity (red) ± SEM of three independent experiments; * p<0.05, ** p<0.01. [file 1471-2407-13-147-S1.tiff]

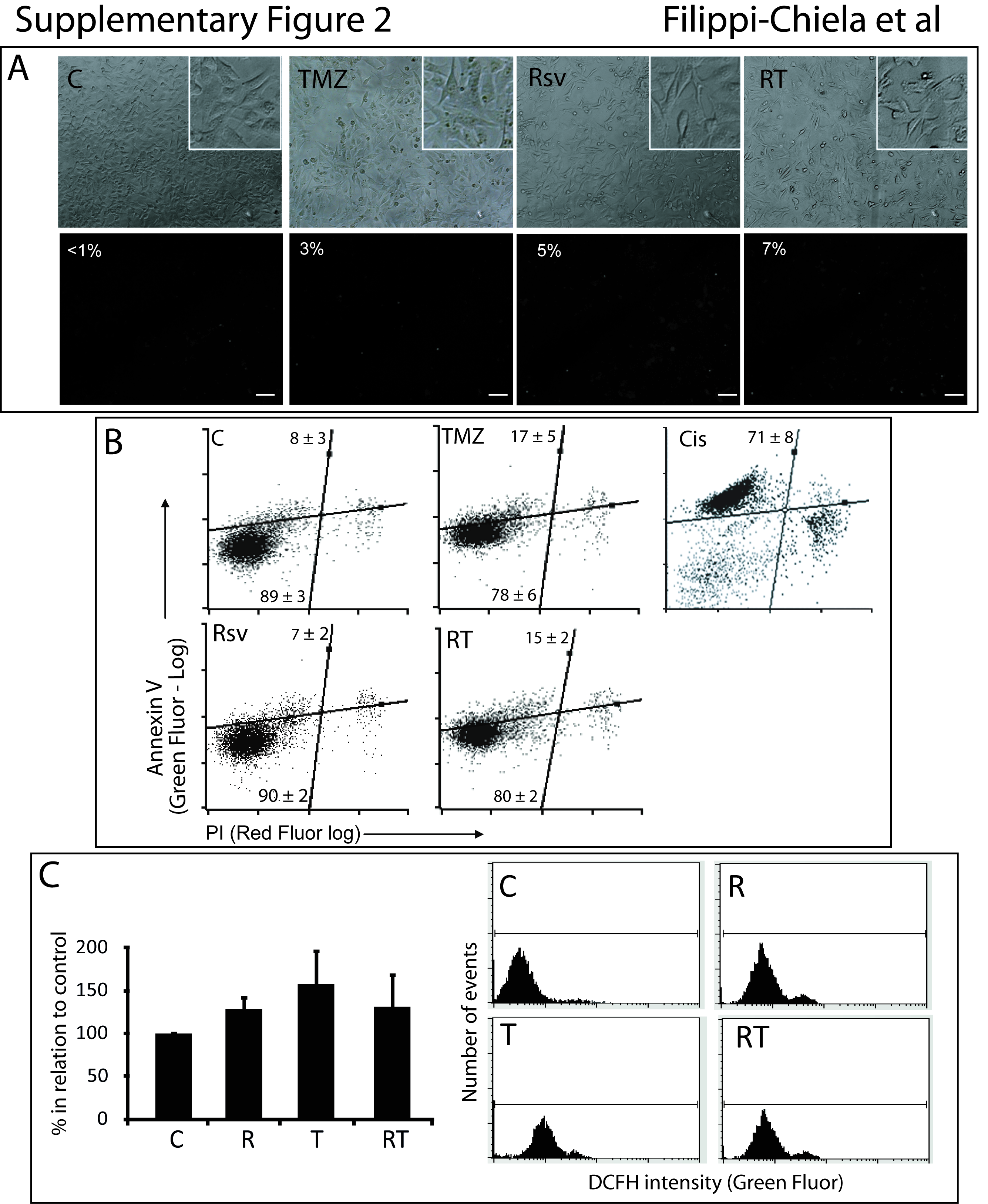

Supplement: Additional file 2: Figure S2 — Apoptosis or ROS are not involved in RT toxicity. U87 cells were treated with Rsv 30 μM, TMZ 100 μM or RT for 48 h. After this, cells were (a) stained with 6 μM PI, to evaluate the membrane integrity and induction of necrosis - numbers indicate the percentage of positively marked cells (ratio of PI labeled cells/total cells) for at least 100 cells counted per treatment; scale bar: 100 μm; detail shows the morphology of treated cells. (b) Cells were treated as in (a) and marked with annexin V-FLUOS/PI and evaluated by flow cytometry. Numbers in quadrants represents the percentage of cells ± SEM of three independent experiments; (c) Cells were treated as in (a) and marked with DCFH to measure reactive oxidative species followed by flow cytometry. Left -% in relation to control of DCFH intensity; right – graphs of DCFH staining from Guava Software; n=2. [file 1471-2407-13-147-S2.tiff]

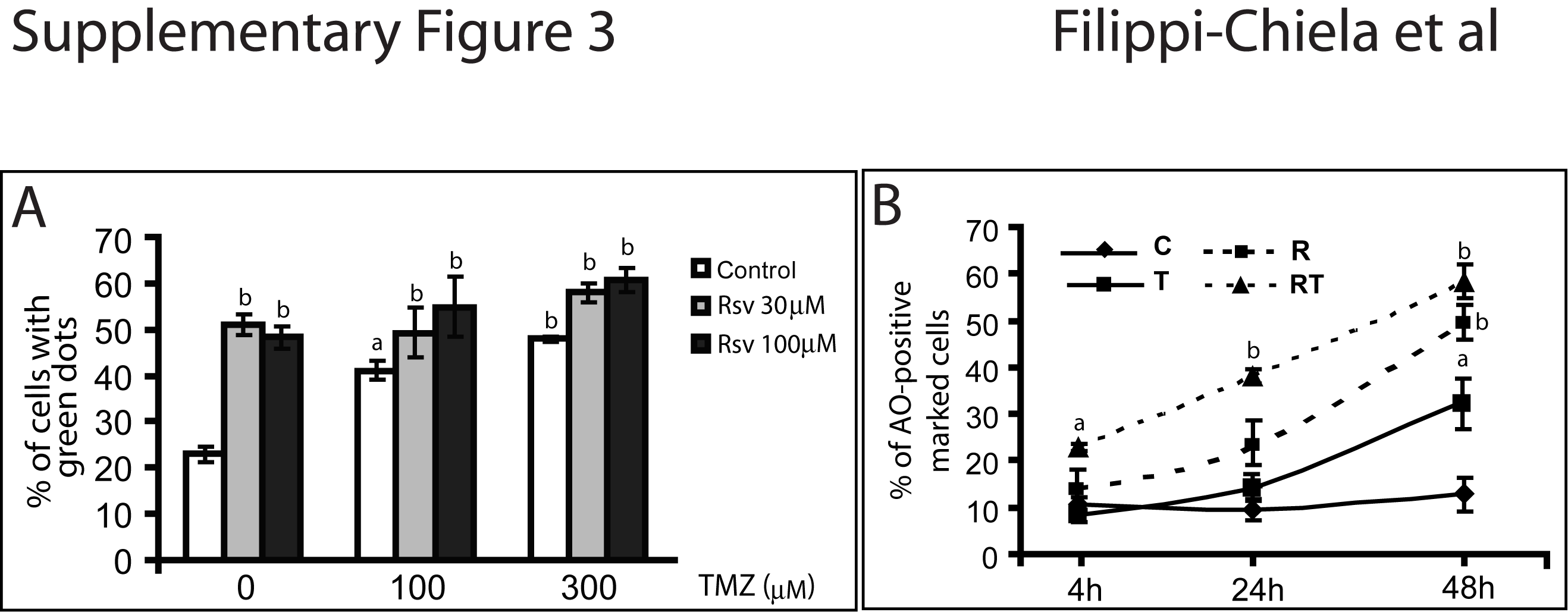

Supplement: Additional file 3: Figure S3 — Autophagy is not directly involved on toxicity of the cotreatment of Rsv and TMZ. (a) Representative images of U87 human GBM cells transfected with the plasmid pRGFP-LC3 and treated with Rsv 30 μM, TMZ 100 μM or R30+T100 for 48 h; white arrows: cytosolic green dots representing LC3-GFP marked autophagosomes; scale bar: 10 μm; (b) percentage of cells that presented more than five well-defined cytosolic green dots, for each treatment; a p<0.01 and b p<0.001 in relation to control; (c) cells were treated as in (A), marked with AO and the percentage of cells positively marked to acridine orange (i.e. red marked cells) were evaluated by flow cytometry after 4, 24 and 48 h; a p<0.05 and b p<0.01 in relation to control. [file 1471-2407-13-147-S3.tiff]

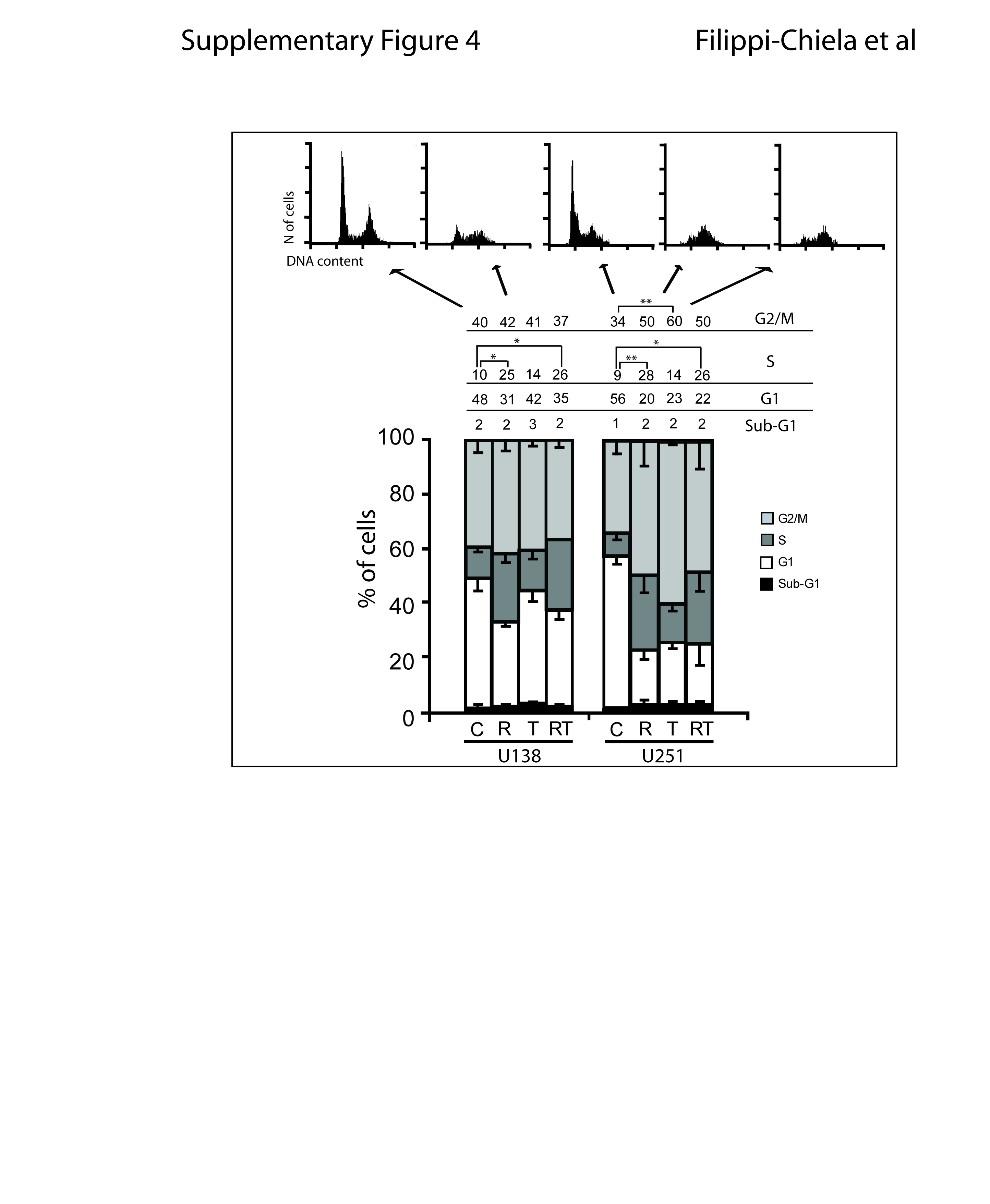

Supplement: Additional file 4: Figure S4 — Rsv abrogates TMZ-induced arrest in G2/Min U251 cells, while U138 is resistant to TMZ-induced arrest. Cell cycle distribution of U251 and U138 cells treated with Rsv 30 μM, TMZ 100 μM or RT, for 48 h. Representatives histograms are shown on the top of figure of flow cytometry; numbers between the lines indicates the percentage of cells in each phase of cell cycle, as indicated; cells were treated as cited above, followed by fixation as described on material and methods section, staining with 6 μM PI and flow cytometry to determination of DNA content; * p<0.05; ** p<0.01. [file 1471-2407-13-147-S4.tiff]
